# Commentary: Do the Benefits of Male Circumcision Outweigh the Risks? A Critique of the Proposed CDC Guidelines

**DOI:** 10.3389/fped.2015.00088

**Published:** 2015-10-14

**Authors:** Brian J. Morris

**Affiliations:** ^1^School of Medical Sciences, University of Sydney, Sydney, NSW, Australia

**Keywords:** evidence-based policy, risk-benefit, preventive medicine, anti-circumcision, ethics

A recent article by ethicist Brian Earp (1) criticizing draft recommendations by the Centers for Disease Control and Prevention (CDC) on the topic of male circumcision (MC) (2) is seriously flawed. Earp seems most concerned about the preservation of the foreskin at all costs. His claims that the foreskin protects the glans and has properties important for sexual pleasure are fallacious. A recent rigorous systematic literature review of penile structures found that sexual pleasure involves neuroreceptors in the glans, not the foreskin (3). It further found that sexual pleasure is derived from exposure of the glans by circumcision or, in uncircumcised men, by foreskin retraction during erection (3). The density of Meissner’s corpuscles in the foreskin *decreases* at puberty and free nerve endings do not correlate with sexual response (3). Research found that the foreskin was ranked last for sexual pleasure and for the density and size of sensory nerve endings when compared with eight other hairless skin types of the body (4).

A detailed systematic review of all studies rated by quality found circumcision had no adverse effect on sexual function, sensitivity, or sensation (5). This was supported by a recent large UK study (6). A large randomized controlled trial found, if anything, sex for men and their female partners was better after circumcision (7). A meta-analysis of all sexual dysfunctions found these did not differ in frequency between circumcised and uncircumcised men (8).

Earp uses as support for his views an opinion piece that was shown to be seriously flawed (9). Those authors subsequently reported finding women preferred circumcised men for sexual activity (10). Their survey also included 28 homosexual men, finding an overall preference for uncircumcised partners, consistent with engagement in foreskin-related sexual activities, such as “docking,” that require the male homosexual partner to be uncircumcised.

Earp assumes that benefits of circumcision do not begin until the boy is older and becomes sexually active. But evidence enunciated by the CDC after its detailed systematic review of the literature shows neonatal circumcision confers immediate benefits. These include protection against urinary tract infections that in infancy, in particular, can cause permanent kidney damage and (rarely) death (11). Other protections in infancy, childhood, and throughout life include reduction in inflammatory skin conditions, phimosis, paraphimosis, foreskin trauma, inferior hygiene, and smegma, especially in circumstances when bathing is infrequent or washing after sexual intercourse does not usually take place (12). Neonatal circumcision also avoids the need for later circumcision, for which risk of adverse events is higher (12). Failure to circumcise early in life usually means it will not happen, even if the male wishes he were circumcised (13). Protection against prostate (14) and penile (15) cancer is greater if circumcision occurs prior to sexual debut.

Earp seems unaware that findings in sub-Saharan Africa of protection against various sexually transmitted infections (STIs), including HIV, during heterosexual intercourse have also been observed in developed countries, such as the USA, the UK, and Australia (12, 16, 17). Although Earp is correct in pointing to sexual intercourse amongst homosexual men as being the primary source of HIV infections in most developed countries, even in such men, those who engage in insertive, but not receptive, anal intercourse are at reduced risk of HIV and syphilis if circumcised (18, 19).

Although the CDC reported that frequency of adverse events seen immediately after infant circumcision are low (0.2–0.4%), Earp claims that the CDC did not consider long-term adverse events. Based on the evidence, loss of the foreskin is not one of these. Earp refers to meatal stenosis, which was reported in a large CDC study of adverse events in U.S. medical settings from 2001 to 2010 to have been seen in only 0.1% (20). There is no good evidence that meatal stenosis differs in prevalence between circumcised and uncircumcised males. The CDC study found adverse events in general were significantly higher in uncircumcised compared with circumcised boys during infancy. These included, respectively, “reconstruction of penis” (64 vs. 15), overall surgical procedures (224 vs. 52), incision of penis (202 vs. 35), disorders overall (1062 vs. 799), other specified disorders of penis (934 vs. 638), gangrene, death, and decay of body tissue (30 vs. 13) (20).

Earp’s concerns about relative vs. absolute risk reduction are fully accounted for in the risk-benefit analysis (12) cited in the CDC’s report. This analysis presented data on all benefits and risks then calculated a combined figure that found benefits exceed risks by 100 to 1, and that over the lifetime 1 in 2 uncircumcised males will be affected by a medical condition attributable to their foreskin (12). Although most adverse events of circumcision are easily and immediately treatable with complete resolution, the conditions and diseases resulting from lack of circumcision range from ones that can be resolved by treatment (often by circumcision) to ones that can lead to substantial morbidity or even death (12). The latter include STIs and potentially fatal conditions, such as from HIV or penile and prostate cancer, and cervical cancer in the female sexual partners.

He refers to deliberations by a 2009 CDC committee about delaying circumcision so as not to violate autonomy, but fails to cite the report’s conclusion, namely that, “both a decision to circumcise and a decision to not circumcise are legitimate decisions, and either decision is an appropriate exercise of parental authority on behalf of a minor child.”

Earp’s comparison of MC with female genital cutting (which he erroneously terms “female circumcision”) is inappropriate since the latter confers no benefits to health, and especially in its more extreme forms only harms.

Given the benefits of neonatal circumcision and very low risks, neonatal circumcision cannot be considered “unnecessary.” Rather, logic dictates that it would be unethical not to recommend neonatal circumcision. Parents have a legal right to make decisions in the best interest of the health of their children. These include vaccination, healthy diet, shelter, education, and, in the case of males, circumcision. Opponents of MC use as support anecdotes and low quality studies that have generally been the subject of substantial published criticisms. Because of the evidence it is not surprising that comprehensive literature reviews (not cherry-picked studies) by both the CDC and the American Academy of Pediatrics have led to the endorsement of both neonatal and, in the case of the CDC, later age MC. Although debate on any issue is to be welcomed, a reader might wonder about the veracity of arguments by a lone ethicist with a history of opposition to circumcision in questioning the authority of such major health bodies as the CDC and AAP? Unlike opponents, the CDC and AAP used an evidence-based approach involving a thorough review of research findings over many years, giving emphasis to high quality studies. As a result, the conclusions they reached should be regarded as reliable.

## Conflict of Interest Statement

The author declares that the research was conducted in the absence of any commercial or financial relationships that could be construed as a potential conflict of interest.

## References

[1] Earp BD. Do the benefits of male circumcision outweigh the risks? A critique of the proposed CDC guidelines. Front Pediatr (2015) 3:18. 10.3389/fped.2015.00018 25853108 PMC4364150

[2] Centers for Disease Control and Prevention. Recommendations for Providers Counseling Male Patients and Parents Regarding Male Circumcision and the Prevention of HIV Infection, STIs, and Other Health Outcomes. Docket No. CDC-2014-0012 (2014). Available from: http://www.gpo.gov/fdsys/pkg/FR-2014-12-02/pdf/2014-27814.pdf

[3] Cox G Krieger JN Morris BJ. Histological correlates of penile sexual sensation: does circumcision make a difference? (Systematic review). Sex Med (2015) 3:76–85. 10.1002/sm2.67 26185672 PMC4498824

[4] Bhat GH Bhat MA Kour K Shah BA Density and structural variations of Meissner’s corpuscles at different sites in human glaborous skin. J Anat Soc India (2008) 57:30–3.

[5] Morris BJ Krieger JN. Does male circumcision affect sexual function, sensitivity or satisfaction? – A systematic review. J Sex Med (2013) 10:2644–57. 10.1111/jsm.12293 23937309

[6] Homfray V Tanton C Mitchell KR Miller RF Field N Macdowall W Examining the association between male circumcision and sexual function: evidence from a British probability survey. AIDS (2015) 29:1411–6. 10.1097/QAD.0000000000000745 26091302 PMC4502984

[7] Krieger JN Mehta SD Bailey RC Agot K Ndinya-Achola JO Parker C Adult male circumcision: effects on sexual function and sexual satisfaction in Kisumu, Kenya. J Sex Med (2008) 5:2610–22. 10.1111/j.1743-6109.2008.00979.x 18761593 PMC3042320

[8] Tian Y Liu W Wang JZ Wazir R Yue X Wang KJ. Effects of circumcision on male sexual functions: a systematic review and meta-analysis. Asian J Androl (2013) 15:662–6. 10.1038/aja.2013.47 23749001 PMC3881635

[9] Morris BJ Krieger JN The literature supports policies promoting neonatal male circumcision in North America. J Sex Med (2015) 12:1305 10.1111/jsm.12855 25757531

[10] Bossio JA Pukall CF Bartley K You either have it or you don’t: the impact of male circumcision status on sexual partners. Can J Hum Sex (2015) 24:104–19. 10.3138/cjhs.242-A2

[11] Morris BJ Wiswell TE. Circumcision and lifetime risk of urinary tract infections: a systematic review and meta-analysis. J Urol (2013) 189:2118–24. 10.1016/j.juro.2012.11.114 23201382

[12] Morris BJ Bailis SA Wiswell TE. Circumcision rates in the United States: rising or falling? What effect might the new affirmative pediatric policy statement have? Mayo Clin Proc (2014) 89:677–86. 10.1016/j.mayocp.2014.01.001 24702735

[13] Morris BJ Waskett JH Banerjee J Wamai RG Tobian AA Gray RH A ‘snip’ in time: what is the best age to circumcise? BMC Pediatr (2012) 12:20. 10.1186/1471-2431-12-20 22373281 PMC3359221

[14] Wright JL Lin DW Stanford JL. Circumcision and the risk of prostate cancer. Cancer (2012) 118:4437–43. 10.1002/cncr.26653 22411189 PMC3376675

[15] Maden C Sherman KJ Beckmann AM Hislop TG Teh CZ Ashley RL History of circumcision, medical conditions, and sexual activity and risk of penile cancer. J Natl Cancer Inst (1993) 85:19–24. 10.1093/jnci/85.1.19 8380060

[16] Morris BJ Hankins CA Tobian AA Krieger JN Klausner JD. Does male circumcision protect against sexually transmitted infections? Arguments and meta-analyses to the contrary fail to withstand scrutiny. ISRN Urol (2014) 2014(684706):1–23. 10.1155/2014/684706 24944836 PMC4040210

[17] Chemtob D Op de Coul E van Sighem A Mor Z Cazein F Semaille C. Impact of male circumcision among heterosexual HIV cases: comparison between three low prevalence countries. Isr J Health Policy Res (2015) 4:36. 10.1186/s13584-015-0033-8 26244087 PMC4524174

[18] Templeton DJ Jin F Prestage GP Donovan B Imrie JC Kippax SC Circumcision and risk of sexually transmissible infections in a community-based cohort of HIV-negative homosexual men in Sydney, Australia. J Infect Dis (2009) 200:1813–9. 10.1086/648376 19911990 PMC2784211

[19] Templeton DJ Jin F Mao L Prestage GP Donovan B Imrie J Circumcision and risk of HIV infection in Australian homosexual men. AIDS (2009) 23:2347–51. 10.1097/QAD.0b013e32833202b8 19752714

[20] El Bcheraoui C Zhang X Cooper CS Rose CE Kilmarx PH Chen RT. Rates of adverse events associated with male circumcision in US medical settings, 2001 to 2010. JAMA Pediatr (2014) 168:625–34. 10.1001/jamapediatrics.2013.5414 24820907 PMC4578797

